# Correction to: Renal complications in coronavirus disease 2019: a systematic review

**DOI:** 10.1186/s41232-021-00171-w

**Published:** 2021-07-23

**Authors:** Taichiro Minami, Yasunori Iwata, Takashi Wada

**Affiliations:** 1 Renal Division, Department of Medicine, Brigham and Women’s Hospital, 4 Blackfan Street, Boston, MA USA; 2 Department of Nephrology and Laboratory Medicine, Kanazawa University, Kanazawa, Ishikawa Japan

**Correction to: Inflamm Regener 40, 31 (2020)**

**https://doi.org/10.1186/s41232-020-00140-9**

Following publication of the original article [1], the authors reported an update in reference number 40.

The original reference number 40 is:

- 40. Kashi AH, Fallah-Karkan M, Amini E, Vaezjalali M. The presence of COVID-19 in urine: a systematic review and meta-analysis of the literature. medRxiv. 2020. 10.1101/2020.05.15.20094920.

The updated reference number 40 is:

- 40. Kashi AH, De la Rosette J, Amini E, Abdi H, Fallah-Karkan M, Vaezjalali M. Urinary Viral Shedding of COVID-19 and its Clinical Associations: A Systematic Review and Meta-analysis of Observational Studies. Urol J. 2020 Sep 5;17(5):433–441. doi: 10.22037/uj.v16i7.6248. PMID: 32888186.

The original article [1] has been updated.
